# Developing of a new guideline for improving birth experiences among Iranian women: a mixed method study protocol

**DOI:** 10.1186/s12978-020-0868-5

**Published:** 2020-01-30

**Authors:** Solmaz Ghanbari-Homayi, Zahra Fardiazar, Sakineh Mohammad-Alizadeh-Charandabi, Mohammad Asghari Jafarabadi, Eesa Mohamadi, Shahla Meedya, Mojgan Mirghafourvand

**Affiliations:** 1 Department of Midwifery, Faculty of Nursing and Midwifery, Tabriz University of Medical Sciences, Tabriz, Iran; 2 Women Reproductive Health Research Center, Tabriz University of Medical Sciences, Tabriz, Iran; 3 Social determinants of Health Research Center, Tabriz University of Medical Sciences, Tabriz, Iran; 4 Department of Statistics and Epidemiology, Tabriz University of Medical Sciences, Tabriz, Iran; 5 Road Traffic lnjury Research Center, Tabriz University of Medical Sciences, Tabriz, Iran; 6 Department of Nursing, School of Medicine, Tarbiat Modares University, Tehran, Iran; 7 South Asia Infant Feeding Research Network (SAIFRN), School of Nursing, Faculty of Science, Medicine and Health, University of Wollongong, Wollongong, Australia

**Keywords:** Childbirth, Birth experience, Primiparity, Support, Control, Guideline, Mixed method

## Abstract

**Background:**

The childbirth experience has significant effects on the life of the mother and family. However, there are no Iranian studies which evaluate and measure women’s childbirth experiences to provide accurate data on this important matter. The aim of this study is to develop a new guideline to improve women’s childbirth experiences by meeting their needs and expectations.

**Methods/design:**

The present study will use the mixed method with the explanatory sequential approach. Phase one is a cross-sectional survey with random cluster sampling of the health centers in Tabriz. Eight hundred primiparous women will be selected to measure their childbirth experiences and predictors factors. Phase two is a qualitative study to explore women’s perceptions of the aspects and determinants of the childbirth experience. Phase two participants will be selected using purposive sampling from the women who participated in phase one. Phase three involves developing a new guideline to improve women’s childbirth experiences. The new guideline will be developed based on the following elements: a) the results of the qualitative and quantitative data from phase one and two, b) a review of the related literature, and c) expert opinions that have been collected using the Delphi technique.

**Discussion:**

By exploring women’s childbirth experiences and the influencing factors, a culturally sensitive evidence-based guideline can be developed. The provision of the evidence-based guideline resulting from this study might be effective in improving the quality care of the services for pregnant women.

**Ethical code:**

IR.TBZMED.REC.1396.786.

## Plain English summary

The first childbirth experience can affect not only a broad range of personal and social aspects of a woman’s life in the future but also her perception of the future childbirth experience. Furthermore, women with a negative birth experience are more likely to request a cesarean. Asking for a C-section without indication can lead to an increased rate of negative childbirth experience.

Although childbirth is a global event and some approaches to improve the childbirth experience have been adopted in the majority of developed countries, the childbirth experience is a momentous mental and personal concept. The childbirth experience differs from one woman to another and is affected by internal, cultural, and social learning factors. There are currently no Iranian studies which accurately evaluate and measure women’s childbirth experiences using standard validated tools. The current study is designed to develop a new guideline to improve women’s childbirth experiences.

The present study will use a mixed method with an explanatory sequential approach. This study will have three phases. Phase one is divided to two sections: the aim of first section is to adapt the scales to the Iranian context and determine their psychometric characteristics. The second section is a descriptive-analytic cross-sectional study to identify the women’s childbirth experiences and influencing factors. Phase two is an exploratory qualitative study to explore women’s childbirth experiences in more detail. Phase three is about developing an evidence based and culturally sensitive guideline based on literature review, the results of phase one and two and experts’ opinion using the Delphi method.

## Background

Childbirth experience is defined as an “individual life event, incorporating interrelated subjective psychological and physiological processes, influenced by social, environmental, organizational and policy contexts” [1]. Traditionally, maternity health care services focused their efforts and resources on reducing perinatal mortality and paid less attention to mothers’ childbirth experiences and beliefs about motherhood [2, 3]. However, women’s experiences of the childbirth process are long-term memories [4] and can influence their mental health status [5]. Negative childbirth experiences are associated with post-traumatic stress disorder (PTSD) [6], poor mother-infant attachment [7], sexual dysfunction, mood and behavior disorder [8], postnatal depression [9], and decisions about future pregnancies [10]. Furthermore, women with a negative birth experience are more likely to request a cesarean [11]. This is a more common issue in Iran. The prevalence of cesarean section in Iran is estimated at 48% [12] and a previous negative childbirth experience as a non-medical factor is one of the most important reasons Iranian women request cesarean sections [12]. Asking for a C-section without indication can lead to an increased rate of negative childbirth experience. Moreover, women undergoing a C-section displayed a more negative attitude towards themselves and their infants. They also demonstrated poorer parental behaviours and were more prone to postpartum mood disorders [13–15].

The first childbirth experience can affect not only a broad range of personal and social aspects of a woman’s life in the future [8] but also her perception of the future childbirth experience. In that, some studies have shown that the childbirth experience of multiparous women depends on their first childbirth experience [16].

Women’s childbirth experiences and influencing factors are well known in developed countries [14, 16–21]. According to the findings of these studies, factors such as instrumental childbirth [17], fear of childbirth [18], complications during pregnancy [19], previous negative birth experience [16, 17], perceived poor support and control [20, 21], neonatal complications [14] are well known risk factors of a negative birth experience.

Developed countries have developed and applied standard scales for an accurate measurement of women’s childbirth experience. More than 30 standard scales for assessing the experience of childbirth have been introduced, and almost all are based on the experiences of women in developed countries. Some standard scales commonly used in research include “The Labor Agent Scale (LAS)”, “The Wijma Delivery Expectancy / Experience Questionnaire (W-DEQ”, “The Childbirth Perception Scale”, “The Childbirth Trauma Index (CTI)”, and “Childbirth Experiences Questionnaire (CEQ)” [22].

However, there are only a limited number of developing countries that have investigated the childbirth experience. Results of a descriptive study in Turkey on primiparous women showed that they received many midwifery interventions during labour in both public and private hospitals. In addition, they rarely received food during labour and had no skin-to-skin contact with their infants. They also experienced a high level of fear and anxiety. Moreover, the medical staff helped these women only for body movements and position change [23]. A qualitative study with a phenomenological approach was conducted on childbirth experience and its meaning among women in Uganda. The mothers in this study considered childbirth pain as a natural phenomenon from a social perspective. Providing mothers with physical, mental, and social support, peace, sympathy, and encouragement resulted in a positive childbirth experience; whereas, inappropriate care, poor communication, and the lack of privacy led to a negative maternal childbirth experience [24]. A qualitative study with a content analysis approach explained the childbirth experience of women in the west of Kenya. The experience of this group of women was affected by external factors, such as the behaviour of clinical staff, their support, and childbirth facilities and environment [25].

However, there are currently no Iranian studies which accurately evaluate and measure women’s childbirth experiences using standard validated tools. Although childbirth is a global event and some approaches to improve the childbirth experience have been adopted in the majority of developed countries, the childbirth experience is a momentous mental and personal concept [1]. The childbirth experience differs from one woman to another and is affected by internal, cultural, and social learning factors. In that, women’s expectations and preferences during labour and delivery depend on the values of their community. Moreover, the childbirth experience is a complex concept, which includes a broad range of dimensions. The frequency of developed instruments to measure childbirth experience reflects the attempts made to identify and evaluate its complex dimensions [26–28].

Considering that the cultural, economic, and social differences of different societies require examining human experiences in each individual society, there is a need for a rigorous multiphasic study with a mixed method approach to evaluate Iranian women’s childbirth experiences systematically, and to develop an evidence- based guideline to improve their experiences.

### Objectives

The aim of the study is to develop a new guideline to improve women’s childbirth experiences. The following items are the specific objectives of the study.

### The specific objectives are

#### Phase 1

1. To adapt the “CEQ 2.0” and “SCIB” scales to the Iranian context and determine their psychometric properties.
2. To measure the childbirth experience score among primiparous women who gave birth in public and private hospitals of Tabriz, Iran.
3. To determine perceived support and control in birth among primiparous women.
4. To determine the relationship between perceived support and control in birth with childbirth experience among primiparous women.
5. To determine the relationship between sociodemographic characteristics and childbirth experience among primiparous women.
6. To determine the relationship between antenatal, intrapartum factors and childbirth experience among primiparous women.

#### Phase 2

7. To explore women’s perceptions regarding the aspects and determinants of childbirth experience among primiparous women.

#### Phase 3

8. To develop an evidence-based guideline to improve women’s childbirth experience by meeting their needs and expectations.

## Methods/ design

### Study design

The present study will use a mixed method with an explanatory sequential approach. The paradigm of the mixed method is based on the principle of pragmatism. Based on this paradigm, the mixed use of quantitative and qualitative approaches leads to a better understanding of the problem [29, 30].

The sequential explanatory design is a mixed method that firstly collects and analyses quantitative data and then qualitative data to explain and/or generalise quantitative data. In this model, the researcher first identified quantitative data that required further explanation and then collected qualitative data from participants, who could contribute to a deeper explanation of the collected data [30].

**Fig. 1:**
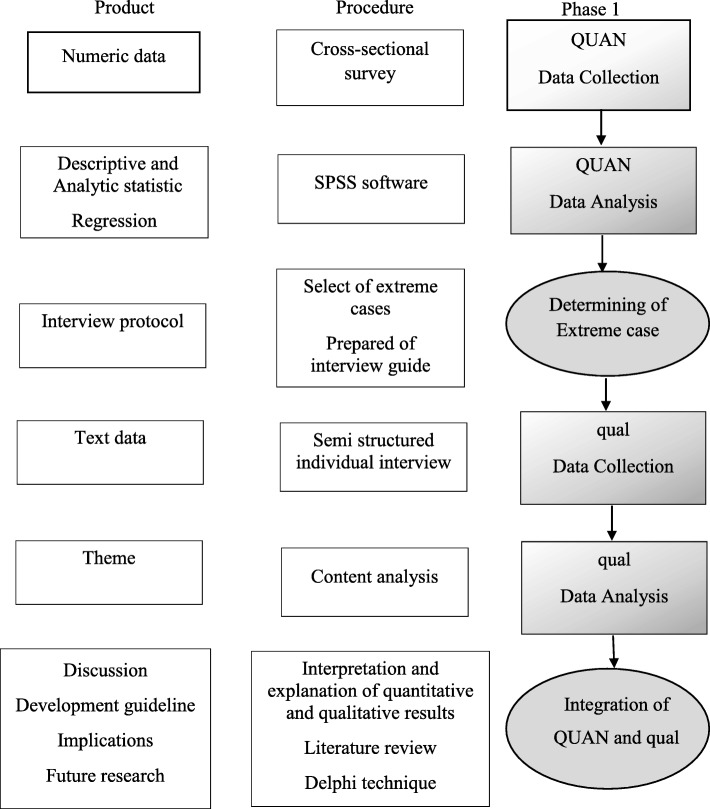
Study visual diagram

This study will have three phases. Phase one is divided to two sections: the aim of first section is to adapt the scales to the Iranian context and determine their psychometric characteristics. The second section is a descriptive-analytic cross-sectional study to identify the women’s childbirth experiences and influencing factors (socio-demographic, antenatal, and intrapartum factors). Phase two is an exploratory qualitative study to explore women’s childbirth experiences in more detail. Phase three is about developing an evidence based and culturally sensitive guideline based on literature review, the results of phase one and two and experts’ opinion using the Delphi method (Fig. 1).

### Phase one: quantitative study

A descriptive-analytic cross-sectional study will assess childbirth experiences and its relationship with support and control in birth, sociodemographic, antenatal and intrapartum factors among the participants. The target population will be primiparous women who give birth in public and private hospitals in Tabriz, Iran. Childbirth Experience Questionnaire 2.0 (CEQ 2.0) and Support and Control in Birth (SCIB) scales will be used to collect the data. The data collection tools will be validated and standardized for use among the Iranian population.

#### Sample size and sampling method

According to Nunnally & Bernstien, for a factor analysis in the validation of scales, 10 samples per item are required [31]. Given the 33 items in SCIB, 330 samples are needed. However, a sample of 660 people is required due to the sampling plan and the design effect of 2. Considering that the sampling plan in the first phase is cluster, the design effect must be considered. “The loss of effectiveness by the use of cluster sampling, instead of simple random sampling, is the design effect” [28]. Using a cluster sample usually needs a larger sample size than a simple random sampling and for a well-designed study, the design effect usually ranges from 1 to 3 [32, 33]. Considering a minimum 20% attrition rate, the total required sample size is calculated to be a minimum of 800 participants.

First, the rate of vaginal childbirth in public and private hospitals will be extracted separately. Then, all of the urban and suburban Tabriz health centers will be listed and numbered (114 centers). Using cluster sampling and the website www.random.org, approximately half of the centers will be randomly selected. A list of potential participants will be arranged based on the health records at each selected center. From the list, the required sample size for each center will be determined using the proportional sampling method by the rate of vaginal childbirth in public and private hospitals and the samples will be randomly selected. Using the phone number registered in each record, the researcher will call the potential participants and invite them to participate in the study.

The eligible participants will be provided with full explanations about the study objectives and procedures. Socio-demographic questionnaire, Childbirth Experience Questionnaire 2.0 (CEQ 2.0), and Support and Control in Birth questionnaire (SCIB) will be filled out in a relatively quiet environment by a research staff member and collected in person. A number of participants are likely to be illiterate, so the questionnaires will be completed by the researcher in order for the data collection method to be the same for all individuals. Obstetrics information will be collected by referring to individuals’ medical records. Also, informed consent will be obtained before collecting information from women’s medical records.

#### Inclusion criteria

The inclusion criteria for women are being Iranian, residing in Tabriz, and being at least 18 years old with normal mental or physical status and primiparous who gave birth to a singleton full term neonate.

#### Exclusion criteria

The exclusion criteria for women are having mental disability; being deaf or mute; taking any antidepressant medication e.g. tricyclic antidepressants, serotonin reuptake inhibitors, monoamine oxidase, serotonin-norepinephrine reuptake inhibitor; having stressful incidents during the last 3 months such as divorce, death of first-degree family members, diagnosis of a hard-to-treat or incurable disease of a family member; multiple pregnancy; preterm delivery (less than 37 weeks of gestation); post-term delivery (over 42 weeks of gestation); non-cephalic presentation; emergency cesarean section; history of depression or postpartum depression; obstetric problems such as placenta previa, placental abruption, preeclampsia, neonatal with major malformations, and death of the newborn. Furthermore, if the participants become exposed to any stressful incident during the study, they will be excluded from the study.

#### Scales and data collection

Quantitative data are collected using sociodemographic and obstetric characteristics questionnaires, CEQ 2.0 and SCIB by a research staff member and collected in person. Sociodemographic and obstetric questionnaires will include questions on age, spouse’s age, and medical interventions during labor such as induction and augmentation, epidural analgesia, immobilization in labor.

The second version of CEQ contained 23 items and measures the women’s perceptions of first labour and birth, and includes the following subscales: own capacity (personal feeling about childbirth and labor pain), professional support (information and midwifery care), perceived safety (sense of safety and childbirth memories), and participation (the person’s ability to change childbirth situation). The CEQ consists of 20 items in the form of multiple-choice questions and three items completed using Visual Analog Scale (VAS). Participants will be asked to rate their level of agreement or disagreement with each item by using a 4 point Likert scale where 1 = totally agree; 2 = mostly agree; 3 = mostly disagree; and 4 = totally disagree. Questions answered in the VAS form will be changed from a 0–100 score to 1–4 score where scores 0–40 = score 1, scores 41–60 = score 2, scores 61–80 = score 3, and scores 81–100 = score 4. Statements with negative concepts (experiencing severe pain, feeling tired, scared and having a bad memory) are scored in reverse. Higher mean scores reflect more positive childbirth experiences. The overall Cronbach’s alpha of the original CEQ has not been reported. However, the Cronbach’s alpha for own capacity, participation, perceived safety, and professional support subscales has been reported 0.82, 0.62, 0.78, and 0.88 respectively [34]. This tool will be completed at least 1 month after childbirth.

SCIB contains 33 items and its subscales include internal control, external control, and support. The internal control contains 10 items that focus on the emotions, behavior, pain, and physical functioning (e.g. “I overcame my pain”). The external control contains 11 items that focus on pain relief, information, environment, decisions and procedures, and birth outcome (e.g. “I had control over when procedures happened”). The support contains 12 items that focus on coping methods, staff attitude, empathy and understanding, encouragement, listening, informational support, and support with pain relief (e.g. “The staff dismissed things I said to them”). Questions are scored on a 5-point Likert scale from agree completely to disagree completely. A score of 1 shows less control and support, and a score of 5 shows more control and support. Ten items are scored in reverse. SCIB will be completed 1 month after childbirth. The overall Cronbach’s alpha of the original SCIB has been reported 0.95. Also, the Cronbach’s alpha for internal control, external control, and support subscales has been reported 0.86, 0.93, and 0.93 respectively [21].

Due to the fact that the CEQ 2.0 and SCIB have not been validated in Iran, they will be standardized and validated prior to the start of the main study. The scales will be translated into Farsi using the Forward & Backward Translation method. The translated versions will be edited by two experts in concepts and the final versions will be created. For quantitative valuation of face validity, 20 postpartum mothers will complete the face validity checklist and will be asked to comment on clarity, simplicity, and relevance of items. Based on these notes, the translated items will be revised. The mothers will also be requested to comment on the impact score of the items on a 4-point Likert scale. To obtain the impact score, the percentage of mothers who score the items a value of 4 will be multiplied by the mean total impact score. Impact scores greater than 1.5 will be considered satisfactory [35].

To measure content validity, 10 experts in obstetrics and gynecology, midwifery, psychology, nursing, health promotion and reproductive health, will be invited to comment on transparency, relevance and simplicity of items (CVI = Content Validity Index) and the necessity of items (CVR = Content Validity Ratio). Responses will be scored based on the 4-point Likert scale [“inappropriate” (1); “need to revise” (2); “appropriate but need to modify” (3); and “completely appropriate” (4)]. A CVI greater than 0.79 and CVR greater than 0.62 will be considered satisfactory [36].

#### Data analysis

The data obtained in the first section of phase one will be analyzed using SPSS Statistics Version 25.0 for Windows (IBM Inc., Armonk, NY, USA). In the cross sectional stage, descriptive statistics will be used to describe the socio-demographic, antenatal and intrapartum factors and childbirth experience. The analytical statistics including univariate will be used to test the correlation between socio-demographic, antenatal and intrapartum variables with the childbirth experience. Then, variables with a correlation of *p* < 0.1 in the univariate analysis will be entered into the multivariable logistic model. All tests will be two-tailed. *P*-value <0.05 will be considered statistically significant. To promote data quality in the cross sectional stage, procedures such as double data entry and range checks for data values will be conducted.

The data obtained in the second section of phase one will be analyzed using SPSS Statistics Version 25.0 for Windows (IBM Inc., Armonk, NY, USA) and AMOS software. Construct validity will be measured using exploratory factor analysis and confirmatory factor analysis. In exploratory factor analysis, the Principal Axis Factoring method and oblimin rotation will be used to extract and rotate the factors. If the items loading are lower than 0.3, the item may be removed [37]. Next, exploratory analysis, confirmatory factor analysis will be used to support the findings of the scale dimensions. Indicators with acceptable values for the model to be approved include Root Mean Square Error of Approximation (RMSEA) < 0.08, Goodness of Fit Index (GFI), Comparative Fit Index (CFI) ≥ 0.90, and Normed chi-square ( x^2^/df < 5). Internal consistency will be assessed using the Cronbach’s Coefficient alpha. The test-retest reliability will be calculated through test-retest of 20 primiparous mothers with a 2 week interval and the calculation of Intra Correlation Coefficient (ICC).

### Phase two: qualitative study

Phase two is an exploratory qualitative study with a conventional content analysis approach to explore women’s childbirth experiences in more detail.

#### Sample size and sampling method

Purposeful sampling will be used in phase two. The participants in this phase will be selected based on the mean total score of childbirth experience, which is collected in phase one of the study. Women with 10% upper and lower scores of childbirth experience will be selected as the extreme cases. We will seek to interview women with either a positive or negative childbirth experience to collect more comprehensive information about their childbirth experience and its effective factors.

The analysis process will be initiated from the first interview. The aim is to clarify the details and relationships between the pivotal concepts and categories derived from investigating the initial data. In this way, the selection of the participants will continue until the relationships between research concepts and variables, i.e. theoretical saturation, are determined [38]. In this study, the sampling will continue until the researcher felt no further information will be collected during data analysis and coding. Nevertheless, some references estimated 12 participants as the minimum number of participants required for a qualitative study [39].

#### Data collection

Qualitative data will be collected using individual in-depth, semi-structured interviews with open questions. Before implementing the qualitative phase, the questions in the interview protocol will be designed based on the findings of the first phase of the study and the literature review on the topic especially in developing countries.

The interviewer will be take notes in addition to the audio recording. If participants refuse to be audio recorded, solely notes will be used for data collection. During the interview, the interviewer will record non-verbal data such as tone of voice, facial expressions, and participants’ positions in a particular sheet with the date and place of the interview. Interviews will be conducted in one session for each person and will take up to 1 h [40]. The interviews will be conducted by the first author under the supervision of the research team, specifically the fifth author who is an expert in qualitative studies.

#### Data analysis

The qualitative data will be organized and managed with MAXQDA 12.13.1. Conventional approach to content analysis will be used in the qualitative phase of the study. This analysis is designed to categorise raw data into different classes or concepts based on valid deductions and interpretations. This process uses inductive reasoning, which presents concepts and classes through a precise investigation of data by the researcher [41].

In qualitative content analysis method, the three approaches; conventional, directed and summative are considered. In this study, qualitative content analysis will be used with a conventional approach. In this approach, data analysis begins by reading the entire text repeatedly to obtain a complete understanding of them. Then, texts are read word by word to extract codes by first underlining the exact words from the text that seem to obtain key concepts. Next, the text is made by the notes of first thoughts and initial analysis. Labels for codes emerge that are reflective of more than one key thought. These codes are then classified according to their differences and similarities. Ideally, 10 to 15 categories are sufficient for classifying a large number of codes. The main advantage of conventional content analysis is to obtain direct information from the study without imposing a predetermined category or theory. One challenge facing this type of analysis is its interference with other qualitative approaches, such as the grounded theory and phenomenology. Although these methods are similar in the initial analysis, they are prioritized over contractual content analysis for theory development. It is also useful for model development.^35^ In order to ensure trustworthiness of findings in the qualitative section, four criteria (credibility, reliability, portability and verifiability) were considered [42].

### Phase three: integration of quantitative and qualitative data/ and development of the guideline

In this phase the supportive evidence-based guideline for improving childbirth experiences among women will be developed by using a combination of literature review, the findings of two prior study phases and comments of experts.

To develop a guideline for the improvement of the childbirth experience, the research team will formulate the guideline initially through a comprehensive review of available evidence. The systematic review studies and interventional studies will be conducted to find approaches provided in available guidelines. Search will be done in the English language databases (Cochrane Library, MEDLINE, Web of Science, Embase, Scopus, ProQuest) and Persian (Magiran, SID and Barakat) using the keywords “Birth Experience”, “Labour Experience”, “Childbirth Experience”, “Delivery Experience”, “Maternal Experience”, “Mother’s Experience”, “Birth Satisfaction”, “Childbirth Satisfaction”, “Birth Perception”, “Childbirth Perception” from the inception of the databases until the time of search. Search strategy for Web of Science database will be as follows: (“birth experience*” OR “experience of birth*” OR “childbirth experience*” OR “experience of childbirth*” OR “labour experience*” OR “experience of labour*” OR “delivery experience*” OR “experience of delivery*” OR “mother’s experience*” OR “experience of mother*” OR “maternal experience*” OR “birth satisfaction*” OR “childbirth satisfaction*” OR “satisfaction of birth*” OR “satisfaction of childbirth*” OR “birth perception*” OR “perception of birth*” OR “childbirth perception*”) AND (intervention* OR “randomized controlled trial*” OR “RCT*” OR “randomized clinical trial*” OR “systematic review*” OR “meta-analysis*”).

The quality of relevant evidence will be assessed using GRADE, and then the evidence analysis will be done and recommendations will be formulated from the literature review. The recommendations produced will be delivered to the experts in the second round of the Delphi.

The combination of quantitative and qualitative results is usually carried out in three ways: integration into discussion, integration with a matrix or side by side display and transformation. In the present study, the method of integration into discussion will be used. In this method, findings from the qualitative and quantitative analyses are presented in the discussion. Some researchers who use this method present a paragraph that reports the quantitative results and continues the process with a paragraph related to qualitative results. Some others present descriptive quantitative results and then cite a quotation in the same paragraph to confirm the quantitative result. In another method which is not common, the process starts with quantitative findings and then presents a supportive descriptive quantitative finding [30, 43]. Findings from the integration of qualitative and quantitative findings are provided to the experts, as recommendations, in the second round of the Delphi.

Comments of experts will be collected using the Delphi method [44]. In the first round, the expert panel will be selected to participate in the Delphi stage. The experts will be selected purposively from those in the field of clinical service provision to women. All experts in the panel will be female in the fields of midwifery, obstetrics and gynecology, and fertility health. In the second round, the experts’ opinions about the effective factors of positive and negative childbirth experience will be collected using a questionnaire with open-ended items. The opinions of all experts will be coded and categorized. Then, the desired approaches will be obtained after the integration and removal of duplicated items. Other approaches obtained from the literature review and the integration of quantitative and qualitative results will be added to the approaches extracted from the experts’ opinions in the second round. Finally, all approaches will be prepared in the form of a statement in the third round. In this round, the experts will be asked to agree or disagree with each statement in terms of its effects on positive childbirth experience and feasibility/cost-effectiveness. The responses will be scored on a 5-point Likert scale ranging from 0 (completely disagree) to 4 (completely agree). The consensus score in this round will be 70%. The generally disagreed statements will be delivered once again, along with a statistical summary of responses from the previous stage. In the fourth round, the experts will be asked to give their final opinions about the remaining statements in the form of “I agree” and “I disagree.”

## Discussion

The childbirth process is described as a complex, multidimensional and individual experience with important outcomes of a safe birth and gaining favorable experiences during labor and birth. However, psychological outcomes that women experience are frequently ignored [1, 45] which may have a negative impact on women’s attitudes towards vaginal birth; and it may influence their personal life in the future. The present study will provide detailed information on the experiences of a large group of Iranian primiparous women and the factors that are associated with the experiences.

The proposed study has a few strengths. It will fill crucial knowledge gaps in supporting women during childbirth in an Iranian context. Therefore, it is expected to have important clinical implications. The development of an evidence-based multi-method guideline can be used as a starting point for the development of a guideline for maternity care. It is also designed with a mixed method approach; therefore, it supports the integration of different, even contradictory approaches and methods to understand the concepts [29, 30]. Quantitative and qualitative data collection will assist us in bettering our understanding of women’s experiences during childbirth.

This is among a few studies into the childbirth experience that not only explained women’s experience but also will collect expert opinions. In addition to the development of guidelines based on women’s experience and preferences, the method will engage the experts in the process of guideline development and will investigate the feasibility and cost-effectiveness of the recommendations within Iranian culture.

In addition to teaching hospitals, women admitted to private hospitals will be also included in the current study. In Iran, care conditions and protocols depend on the hospital. As a result, the inclusion of patients admitted to both public and private hospitals can produce more accurate and comprehensive results. Assuming that lifestyle and social environments may affect the expectations and preferences of women and their perception of the childbirth experience, women from health centres in both urban and suburban areas will be included.

There are many studies into the evaluation of the childbirth experience immediately and/or within 24–48 h after delivery. This study will investigate this outcome at least 1 month after the delivery. According to the reports, evaluation of the childbirth experience immediately after delivery or in the postpartum room when the mothers’ conditions are not stabilised yet may result in less accurate responses. In addition, women with a healthy neonate may report a false positive childbirth experience [34, 46]. The sampling method is the other strength of this study as the randomized selection of the participants can make the results generalisable.

Although there are various instruments for evaluating the childbirth experience, there are a relatively greater number of studies into the perception of the childbirth experience with only one or limited questions [47–50]. The childbirth experience is a complicated mental concept with various dimensions [1]. Evaluation of the childbirth experience using a standard scale which has been used in different countries is the other strength of the current study [51–53].

This protocol has some weaknesses, including its implementation in only one city within Iran. To minimize this weakness, we will implement the project in an Iranian metropolis, so that its results can be generalised to other similar cities and environments. According to the literature, the childbirth experience of multiparous women can be affected by their previous childbirth experiences [17]. As a result, we include only primiparous women. However, the exclusion of multiparous women is a limitation for generalisation of the results. Regardless of other factors, high-risk women have a more negative childbirth experience [19]. As a result, we decided to include only low-risk women. Therefore, the results cannot be generalised to high-risk women.

The proposed study has a few strengths. The use of standard scale in this study can lead to a more accurate measurement of childbirth experience. Large sample size, random cluster sampling, sample collection from both urban and suburban health centers and women who give birth in public and private hospitals in Tabriz can be considered strengths of the study. Also, the present study has a few limitations. The results will be restricted to the experience of Iranian mothers. This study will be performed on primiparous and low risk women. Therefore, the results will not be generalized to multiparous or high risk women.

The provision of the evidence-based guideline resulting from this study may be effective in improving the quality care of services for pregnant women where they are encouraged to have vaginal births. The results of the study will provide policymakers and health care stakeholders with a standard guideline to improve the experiences of Iranian primiparous women and its possible positive effect on natural birth.

## Data Availability

Not applicable.

## Abbreviations

CEQ 2.0: Childbirth Experience Questionnaire 2.0
CFI: Comparative Fit Index
CVI: Content Validity Index
CVR: Content Validity Ratio
GFI: Goodness of Fit Index
PTSD: Post-Traumatic Stress Disorder
RMSEA: Root Mean Square Error of Approximation
SCIB: Support and Control in Birth
WHO: World Health Organization
